# The worldwide prevalence of the Dupuytren disease: a comprehensive systematic review and meta-analysis

**DOI:** 10.1186/s13018-020-01999-7

**Published:** 2020-10-28

**Authors:** Nader Salari, Mohammadbagher Heydari, Masoud Hassanabadi, Mohsen Kazeminia, Nikzad Farshchian, Mehrdad Niaparast, Yousef Solaymaninasab, Masoud Mohammadi, Shamarina Shohaimi, Alireza Daneshkhah

**Affiliations:** 1 Department of Biostatistics, School of Health, Kermanshah University of Medical Sciences, Kermanshah, Iran; 2 Department of General Surgery, Faculty of Medicine, Kermanshah University of Medical Sciences, Kermanshah, Iran; 3 Lecturer in International Business & Strategy, Faculty of Business & Law, University of Northampton, Northampton, UK; 4 Student Research Committee, Kermanshah University of Medical Sciences, Kermanshah, Iran; 5 Department of Otolaryngology, School of Medicine, Kermanshah University of Medical Sciences, Kermanshah, Iran; 6 Department of Statistics, Razi University, Kermanshah, Iran; 7 Department of Nursing, School of Nursing and Midwifery, Kermanshah University of Medical Sciences, Kermanshah, Iran; 8 Department of Biology, Faculty of Science, University Putra Malaysia, Serdang, Selangor Malaysia; 9 School of Computing, Electronics and Maths, Coventry University, London, UK

**Keywords:** Dupuytren, Prevalence, Meta-analysis

## Abstract

**Background:**

The Dupuytren disease is a benign fibroproliferative disorder that leads to the formation of the collagen knots and fibres in the palmar fascia. The previous studies reveal different levels of Dupuytren’s prevalence worldwide; hence, this study uses meta-analysis to approximate the prevalence of Dupuytren globally.

**Methods:**

In this study, systematic review and meta-analysis have been conducted on the previous studies focused on the prevalence of the Dupuytren disease. The search keywords were Prevalence, Prevalent, Epidemiology, Dupuytren Contracture, Dupuytren and Incidence. Subsequently, SID, MagIran, ScienceDirect, Embase, Scopus, PubMed and Web of Science databases and Google Scholar search engine were searched without a lower time limit and until June 2020. In order to analyse reliable studies, the stochastic effects model was used and the *I*^2^ index was applied to test the heterogeneity of the selected studies. Data analysis was performed within the Comprehensive Meta-Analysis Software version 2.0.

**Results:**

By evaluating 85 studies (10 in Asia, 56 in Europe, 2 in Africa and 17 studies in America) with a total sample size of 6628506 individuals, the prevalence of Dupuytren disease in the world is found as 8.2% (95% CI 5.7–11.7%). The highest prevalence rate is reported in Africa with 17.2% (95% CI 13–22.3%). According to the subgroup analysis, in terms of underlying diseases, the highest prevalence was obtained in patients with type 1 diabetes with 34.1% (95% CI 25–44.6%). The results of meta-regression revealed a decreasing trend in the prevalence of Dupuytren disease by increasing the sample size and the research year (*P* < 0.05).

**Conclusion:**

The results of this study show that the prevalence of Dupuytren disease is particularly higher in alcoholic patients with diabetes. Therefore, the officials of the World Health Organization should design measures for the prevention and treatment of this disease.

## Background

Dupuytren disease is a benign fibroproliferative disorder that results in the formation of collagen knots and fibres in the palmar fascia. The disease was discovered by Felix Plotter in 1614, yet later attributed to Baron Guillaume Dupuytren, a French physician in 1831 [1–3]. Dupuytren disease usually progresses gradually over the years and is irreversible. Dupuytren leads to abnormal flexion of the fingers, involvement of the metocarpophalangeal joints (MCP) and proximal interphalangeal joints (PIP) and can also involve the distal interphalangeal joint (DIP) [3].

In Dupuytren disease, fibrosis usually begins in the palm of the hand and extends to the fingers (the most common involved finger is the ring finger, followed by the little finger, thumb, middle finger and index finger, respectively) [4]. Dupuytren is not usually painful; however, it gradually restricts the movements of fingers in a curved manner by forming firm bands on the palms and fingers [4, 5].

The main cause of Dupuytren is not yet known, but due to the increase in immune cells and associated phenomena in the infected tissue, it is possible that the disease is related to the immune system [5]. However, numerous studies have been conducted on the factors that cause this disease, in which various genetic and environmental factors have been mentioned [4, 6]. Environmental risk factors include alcohol abuse, smoking, hand injuries, aging and intense physical occupations which involve hands [7, 8].

Also, in some diseases, such as hypertension, alcoholism, diabetes, hyperlipidemia, ischemic heart disease, chronic obstructive pulmonary disease (COPD), pulmonary tuberculosis, epilepsy and rheumatoid arthritis (RA), high prevalence of Dupitren has been reported [6–10].

The Dupuytren disease is the most common genetic disorder in connective tissues, and studies have revealed that there is a significant association between the Dupuytren disease, genetics and family history. However, compared to the environmental factors, family history and masculinity have the greatest impact on the disease. The age of onset of Dupuytren disease in people with a positive family history is lower than in patients without a positive family history [11, 12].

Although the disease is not dangerous, it can cause disability in patients. People with Dupuytren face many difficulties including washing, picking up objects, wearing gloves, holding objects with hands, putting hands in pockets, keeping hands straight and pain [13]. These difficulties can reduce the quality of life of the patients [14].

Various studies have been conducted on the prevalence of the Dupuytren disease, which have reported a prevalence of between 0.2 and 56% [15]. However, there is a lack of a comprehensive study with generalized statistics on the prevalence. Considering the significance of this disease and its negative impacts on patients’ quality of life, the present study evaluates the prevalence of the Dupuytren disease in the world by using systematic review and meta-analysis.

## Methods

In this systematic review and meta-analysis study, the SID, MagIran, ScienceDirect, Embase, Scopus, PubMed and Web of Science databases and the Google Scholar search engine were searched for related articles. To access the targeted articles, the following search keywords were used: Prevalence, Prevalent, Epidemiology, Dupuytren Contracture, Dupuytren and Incidence. In addition, all possible combinations of these words have been searched. No time constraints were considered in the search process and all related studies were identified and the information of these studies was transferred to the EndNote X8 bibliography management software. Therefore, all possible related articles published by June 2020 were identified and their information were transferred and analysed. In order to maximize the comprehensiveness of the search, the lists of sources used in all relevant articles found in the above search were manually reviewed.

### Inclusion criteria

The studies that examined the prevalence of the Dupuytren disease in the world, the studies that were observational (non-interventional studies), and studies that their full texts were available were included in our analysis.

### Exclusion criteria

Exclusion criteria were as follows: unrelated studies, studies without sufficient data, duplicate articles and studies with an unclear methodology.

### Selection process

Initially, the studies that were repeated in various searched databases were excluded from this study. Subsequently, a list of the titles of all the remaining articles was prepared. In next step, the eligible articles were selected by evaluating the articles in this list. In next step, the screening was conducted by carefully reviewing the title and abstract of the remaining articles and subsequently, irrelevant articles were removed in accordance with the inclusion and exclusion criteria. In the second stage, the evaluation of the suitability of the studies, the full text of the possible relevant articles remaining from the screening stage was examined based on the inclusion and exclusion criteria, and at this stage, unrelated studies were omitted. To avoid bias, all steps of reviewing sources and extracting data were performed by two reviewers independently. In the case of excluding an article, the reason was documented. In cases where there was a disagreement between the two reviewers, the article was reviewed by a third reviewer.

### Qualitative evaluation

In order to validate and evaluate the credibility of the articles (i.e., methodological validity and results), a checklist related to the type of study was used. STROBE checklists are commonly used to critically evaluate the observational studies such as the present study. The STROBE checklist consists of six general items including title, abstract, introduction, methods, findings and discussion. Some of these items have subitems, and in total this checklist entails 32 items. In fact, these 32 items describe different methodological aspects of a study including title, problem statement, study objectives, type of study, statistical population of the study, sampling method, the appropriate sample size, definition of variables and procedures, data collection tools, statistical analysis methods and findings. Accordingly, the maximum score that can be obtained from the qualitative evaluation in the STROBE is 32 and the cut-off point’s score is 16. Articles with the scores of 16 and above are considered articles with medium or high methodological quality. On the other hand, articles with the score of less than 16 are considered low quality [16]; low-quality articles were excluded from our work.

### Data extraction

The information of all finalized articles included in the systematic review and meta-analysis processes were extracted using a pre-prepared checklist. This checklist included following fields: title of article, name of first author, year of publication, place of study, sample size, prevalence of Dupuytren disease and age.

### Statistical analysis

To evaluate the heterogeneity of the selected studies, the *I*^2^ index was used (heterogeneities were divided into three categories: less than 25% (low heterogeneity), 25–75% (moderate heterogeneity) and more than 75% (high heterogeneity)). In order to test the publication bias and also due to the high volume of samples included in the study, Begg’s test was used at a significance level of 0.1, and corresponding funnel plots were drawn. Statistical sensitivity analysis was conducted to evaluate the effect of individual studies on the final results. In this study, meta-regression was used for additional analysis, to examine the relationship between the prevalence of the Dupuytren disease with the sample size and the year of the study. Data analysis was performed using the Comprehensive Meta-Analysis Software (version 2.0).

## Results

**Fig. 1:**
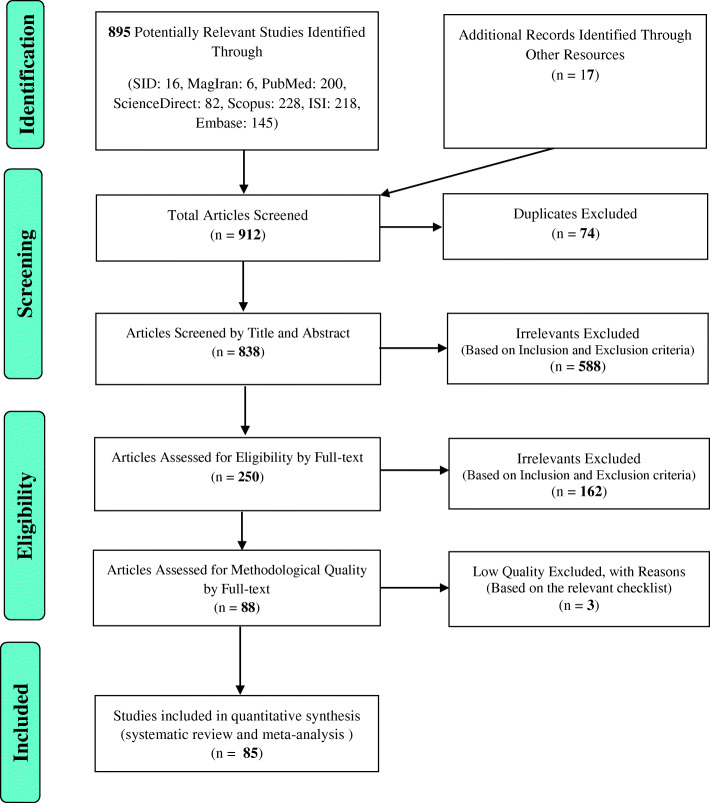
The flowchart on the stages of including the studies in the systematic review and meta-analysis (PRISMA 2009)

The data extracted from the previous studies on the prevalence of the Dupuytren disease were evaluated using a systematic review and meta-analysis. The work has been conducted in accordance with the Preferred Reporting Items for Systematic Reviews and Meta-Analyses (PRISMA) guidelines. Based on the initial search in the databases, 895 potentially related articles were identified and transferred into the EndNote bibliography management software. Seventeen further studies were also added through other sources. Of the total 912 studies identified, 74 were duplicates and were therefore excluded. In the screening phase, out of 838 remaining studies, 588 articles were removed through the study of titles and abstracts and according to the inclusion and exclusion criteria. In the eligibility evaluation stage, out of the remaining 250 studies, 162 articles were omitted due to irrelevance and after examining the full text of the article and similarly by considering the inclusion and exclusion criteria. At the quality evaluation stage, by reviewing the full text of the article and based on the score obtained from the STROBE checklist, out of the remaining 88 studies, 3 studies were excluded due to low methodological quality. These articles obtained the scores of less than 16 based on the STROBE checklist (Fig. 1). Hence, the total number of 85 articles was included in the final analysis stage.

Based on the results from the *I*^2^ 99.9 test and considering the heterogeneity of the selected studies, the random effects model was used to amalgamate the results of the selected studies to approximate the common prevalence. The heterogeneity of the studies could be due to the differences in sample size, sampling error, year of the study or place of study. Of the 85 included articles in systematic review and meta-analysis with a sample size of 6628506 people, 10 studies were conducted in Asia, 56 in Europe, 2 in Africa and 17 in the Americas. The lowest and highest sample sizes were related to the studies of Ardic et al. (*n* = 37) [61] and Nordenskjöld et al. (*n* = 1,300,000), respectively [27]. The characteristics of the included studies in meta-analysis are presented in Table 1.

**Table 1:** Characteristic of included studies prevalence of Dupuytren

Author, year, reference	Age (years)	Country	Sample size total	Sample size male	Sample size female	Prevalence %	Population
**Asia**							
Agrawal et al., 2014, [17]	3783 ≤ 50, 1949 ≥ 50	India	5732	2516	3216	7.2	Diabetic population
Kiani et al., 2014, [18]	54.0 ± 13.2 Fe, 51.6 ± 16.5 Ma	Iran	432	134	298	7.4	Diabetic population
Al-Matubsi et al., 2011, [19]	51.82 ± 11.68	Jordan	187	77	110	17.6	Diabetic population
Lee et al., 2018, [20]	54 men, 49.5 women (mean)	South Korea	16630	-	-	3.2	General population
Rajendran et al. (1), 2010, [21]	53.8 ± 8.2	India	206	98	108	84.0	Diabetic population
Rajendran et al. (2), 2010, [21]	52.4 ± 8.3	India	203	106	97	58.6	General population
Tajica et al., 2014, [22]	66.7 (40–92)	Japan	401	163	238	7.0	General population
Yeh et al., 2015, [23]	60.7 ± 18.4 (men), 53.7 ± 15.5 (women)	China	1078	681	397	8.9	General population
Pandey et al. (1), 2013, [24]	51.8 ± 11.5 (19–65)	India	200	102	98	19.0	Diabetic population
Pandey et al. (2), 2013, [24]	53.1 ± 12.5 (19–65)	India	200	99	101	6.0	General population
**Europe**							
Degreef et al., 2010, [25]	≥ 50	Belgium	500	265	235	31.6	General population
Gumundsson et al., 1999, [26]	46–74	Norway	1297	1297	0	19.2	General population
Nordenskjöld et al., 2017, [27]	Over 20	Sweden	1300000	5208	1299	0.5	General population
Wijnen et al., 2017, [28]	Born between 1900 and 1999	Belgium	215251	451	274	0.3	General population
Arafa et al. (1), 1984, [29]	Over 30	England	392	131	261	6.4	Rheumatoid arthritis
Arafa et al. (2), 1984, [29]	Over 30	England	555	254	301	16.0	General population
Arafa et al. (3), 1991, [30]	Over 30	England	342	177	165	12.0	David Lewis Center Of Epilepsy Patient
Arafa et al. (4), 1991, [30]	Over 30	England	373	241	132	37.8	Chalfont Center Of Epilepsy Patient
Arkkila et al., 1996, [31]	33.2 ± 9.9 type I; 61.1 ± 12.4 type II	Finland	425	200	225	13.9	Diabetic population
Arkkila et al., 2000, [32]	43.4 ± 9.5	Finland	28	28	0	32.1	Diabetes type I
Attali et al. (1), 1987, [33]	54 ± 18	France	432	246	186	28	Different groups which are named
Attali et al. (2), 1987, [33]	58.9 ± 22.7	France	174	77	97	12.6	General population
Bergenudd et al., 1993, [34]	55 years old	Sweden	574	255	319	6.3	Light, moderate and heavy physical workers
French et al., 1990, [35]	23–56	England	50	50	0	6.0	Patients with HIV
Bennett (1), 1982, [36]	15–64	UK	216	216	0	7.4	Manual workers in bagging and packing plant
Bennett (2), 1982, [36]	15–64	UK	84	84	0	1.2	Workers in no bagging or packing
Broekstra et al. (1), 2016, [37]	65–71	Netherlands	169	169	0	51.5	Hockey players aged over 60 years
Broekstra et al. (2), 2016, [37]	59–71	Netherlands	156	156	0	13.5	General population
Burke et al., 2005, [38]	25–99	UK	97537	97537	0	8.1	Miners
Descatha et al., 2012,	20–59	France	2161	2161	0	1.2	Manual workers
Descatha et al., 2013, [39]	59–73	France	13587	3570	1017	7.4	General population
Edington et al. (1), 1991, [40]	62 ± 9	UK	200	124	76	23.5	Diabetic population
Edington et al. (2), 1991, [40]	58 ± 6	UK	170	103	67	24.7	General population
Finsen et al., 2001, [41]	Over 50	Norway	456	261	195	7.7	General population
Gamstedt et al., 1993, [42]	19–62 (mean 42)	Sweden	99	49	50	16.2	Diabetic population
Kristján et al., 2000, [43]	Over 45	Iceland	2165	1297	868	13.3	General population
Carson et al., 1993, [44]	65 to 97 (mean 76.2)	UK	400	400	0	13.8	Ex-military service pensioners
Khan et al., 2004, [45]	Over 40	England and Wales	502493	502493	0	0.03	General population
Kovacs et al. (1), 2012, [46]	52.46 ± 13.56	Romania	187	93	94	26.7	Diabetic population
Kovacs et al. (2), 2012, [46]	51.19 ± 16.21	Romania	197	97	100	5.6	General population
Lanting et al., 2013,	50–89	Netherlands	763	348	415	22.1	General population
Lennox et al., 1993, [47]	Over 60	Scotland	200	100	100	30.0	Consecutive geriatric patients
Noble et al. (1), 1992, [48]	Over 30	England	100			28.0	Alcoholic patients
Noble et al. (2), 1992, [48]	Over 30	England	82			22.0	Hepatic non-alcoholic patient
Noble et al. (3), 1992, [48]	Over 30	England	100			8.0	General population
Palmer et al., 2015, [49]	16–64	UK	4969	4969	0	1.4	General population
Patri et al., 1986, [50]	20–90	France	155	76	79	9.0	General population
Ramchurn et al., 2009, [51]	55	UK	96	60	36	12.5	Diabetic population
Thomas and Clarke, 1992, [52]	25–85	UK	500	499	1	13.6	General population
Caffiniire et al., 1983, [53]	20–65	France	5206	5206	0	3.8	Iron workers
Zerajic and Finsen, 2004, [54]	Over 50	Bosnia and Herzegovina	1207	610	597	25.2	General population
Bulfoni (1), 1980, [55]	-	Italy	125	-	-	72.0	Alcoholic cirrhosis
Bulfoni (2), 1980, [55]	-	Italy	185	-	-	37.8	Hepatic alcoholic involvement without cirrhosis
Bulfoni (3), 1980, [55]	-	Italy	163	-	-	23.9	Alcoholism without hepatic involvement
Diris et al., 2003, [56]	-	France	100	-	-	7.0	Diabetic population
Renard et al. (1), 1994, [57]	-	France	60			35.0	Diabetic type I
Renard et al. (2), 1994, [57]	-	France	60			30.0	Diabetic type II
Renard et al. (3), 1994, [57]	-	France	120			6.7	General population
Stradner et al., 1987, [58]	-	UK	100			42.0	Diabetic population
Trybus et al., 2012, [59]	-	Poland	101			14.9	General population
Sari, 2013, [60]	51.14 ± 15.85	Turkey	21450	6477	14973	0.04	Physiotherapy and rehabilitation
Ardic et al. (1), 2002, [61]	57.8 ± 11.9 (32–81)	Turkey	78	23	55	21.8	Diabetic population
Ardic et al. (2), 2002, [61]	55.7 ± 11.5 (30–79)	Turkey	37	10	27	2.7	General population
Aydeniz et al. (1), 2008, [62]	58.0 ± 9.1	Turkey	102	44	58	12.7	Diabetic population
Aydeniz et al. (2), 2008, [62]	60.1 ± 7.6	Turkey	101	50	51	3.9	General population
Cakir et al., 2003, [63]	46 ± 12 (20–76)	Turkey	137	26	11	8.8	Thyroid disease
**Africa**							
Beighton and Valkenburg, 1974, [64]	Over 35	South Africa	111	63	48	13.5	General population
Mustafa et al., 2016, [65]	57.8 ± 9.5 (range 23–88)	South Africa	1000	478	522	18.6	Diabetic population
**America**							
Barton and Barton (1), 2012, [66]	48 ± 13 M/51 ± 14 W	USA	294	188	106	1.0	Hemochromatosis probands with HFE C282Y homozygosity
Barton and Barton (2), 2012, [66]	48 ± 13 M/51 ± 15 W	USA	67	39	28	1.5	Hemochromatosis probands with C282y/H63d compound heterozygosity
Dibenedetti et al., 2011, [67]	Over 18	USA	23103	11420	11683	2.0	General population
Diep et al., 2015, [68]	Over 18	USA	827	353	474	37.0	Asymptomatic patients
Robert et al., 1977, [69]	29–80 (mean 57)	USA	55	13	42	10.9	Rheumatoid arthritis
Larkin et al., 2014, [70]	52.2 ± 6.9	USA	1217	633	584	8.6	Diabetes type I
Patel et al., 2014, [71]	59.1 ± 12.90	USA	97	51	46	19.6	Patients with psoriasis
Alesia et al., 1999, [72]	Over 25	USA	324300			0.3	General population
Su et al. (1), 1972, [73]	-	USA	142	142	0	12.0	General population
Su et al. (2), 1972, [73]	-	USA	130	130	0	19.2	Alcoholic without cirrhosis
Su et al. (3), 1972, [73]	-	USA	133	133	0	18.0	Alcoholic with cirrhosis
Weinstein et al. (1), 2011, [74]	Over 40	USA	220748	-	-	0.5	Hispanic population
Weinstein et al. (2), 2011, [74]	Over 40	USA	137205	-	-	0.3	Black population
Weinstein et al. (3), 2011, [74]	Over 40	USA	118909	-	-	0.3	White population
Weinstein et al. (4), 2011, [74]	Over 40	USA	70058	-	-	0.3	Asian population
Weinstein et al. (5), 2011, [74]	Over 40	USA	2055	-	-	0.3	Native American
Weinstein et al. (6), 2011, [74]	Over 40	USA	607119	-	-	0.4	Other population

**Fig. 2:**
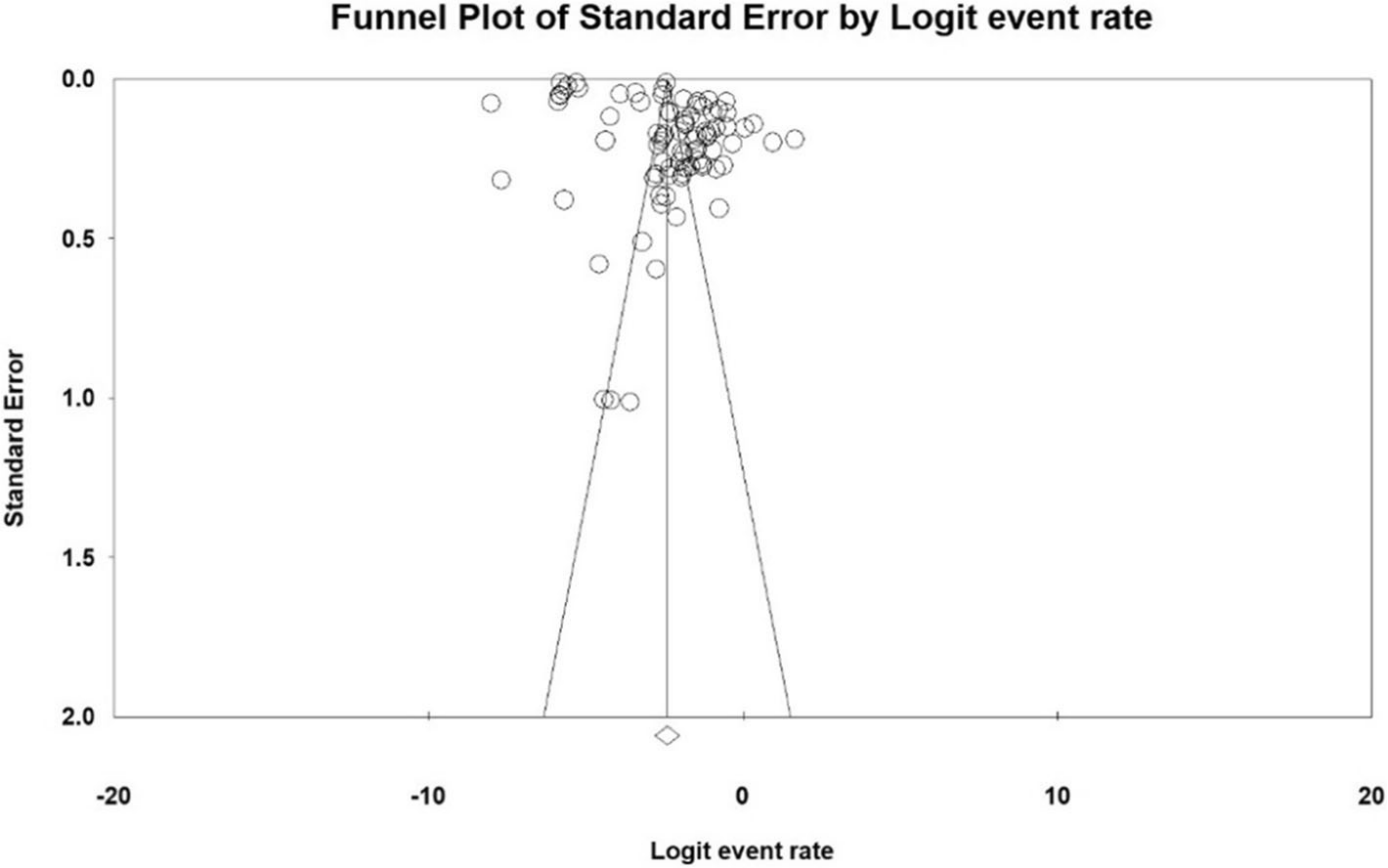
Funnel plot’s results for the prevalence of Dupuytren in the world

The probability of publication bias in the dissemination of the results of Dupuytren disease in the world was performed by Begg’s test (Begg and Mazumdar) at a significance level of 0.1, and through examining the corresponding Funnel plots. The results revealed no publication bias in the present study (*P* = 0.989) (Fig. 2).

**Fig. 3:**
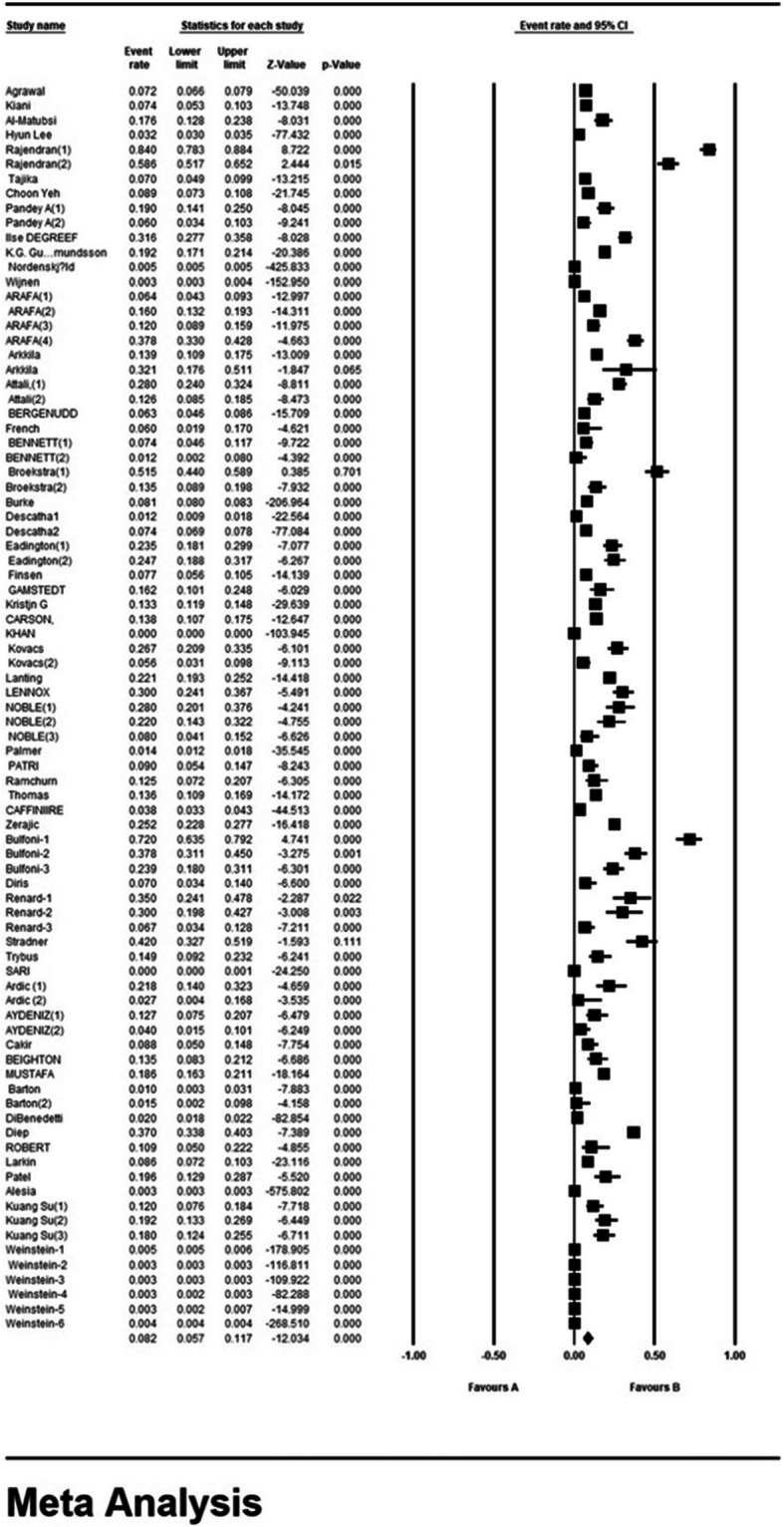
The global prevalence of the Dupuytren disease at 95% confidence interval

According to the results of the present study, the prevalence rate of the Dupuytren disease in the world is 8.2% (95% CI 5.7–11.7%). The midpoint of each segment shows the prevalence in each study and the diamond shape shows the prevalence in the population of the entire studies (Fig. 3).

### Meta-regression test

**Fig. 4:**
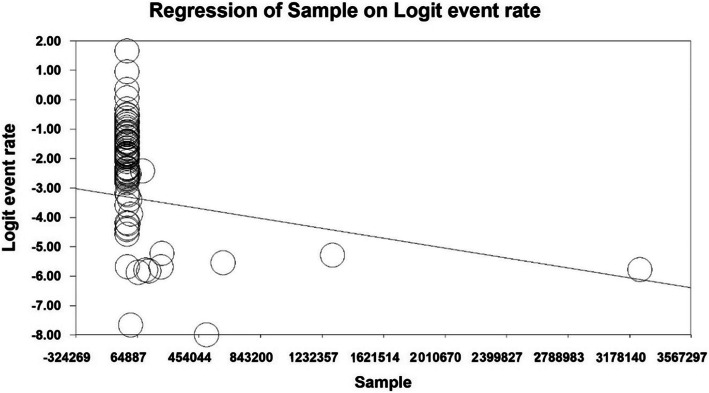
Meta-regression diagram of the prevalence of Dupuytren disease in the world by sample size

**Fig. 5:**
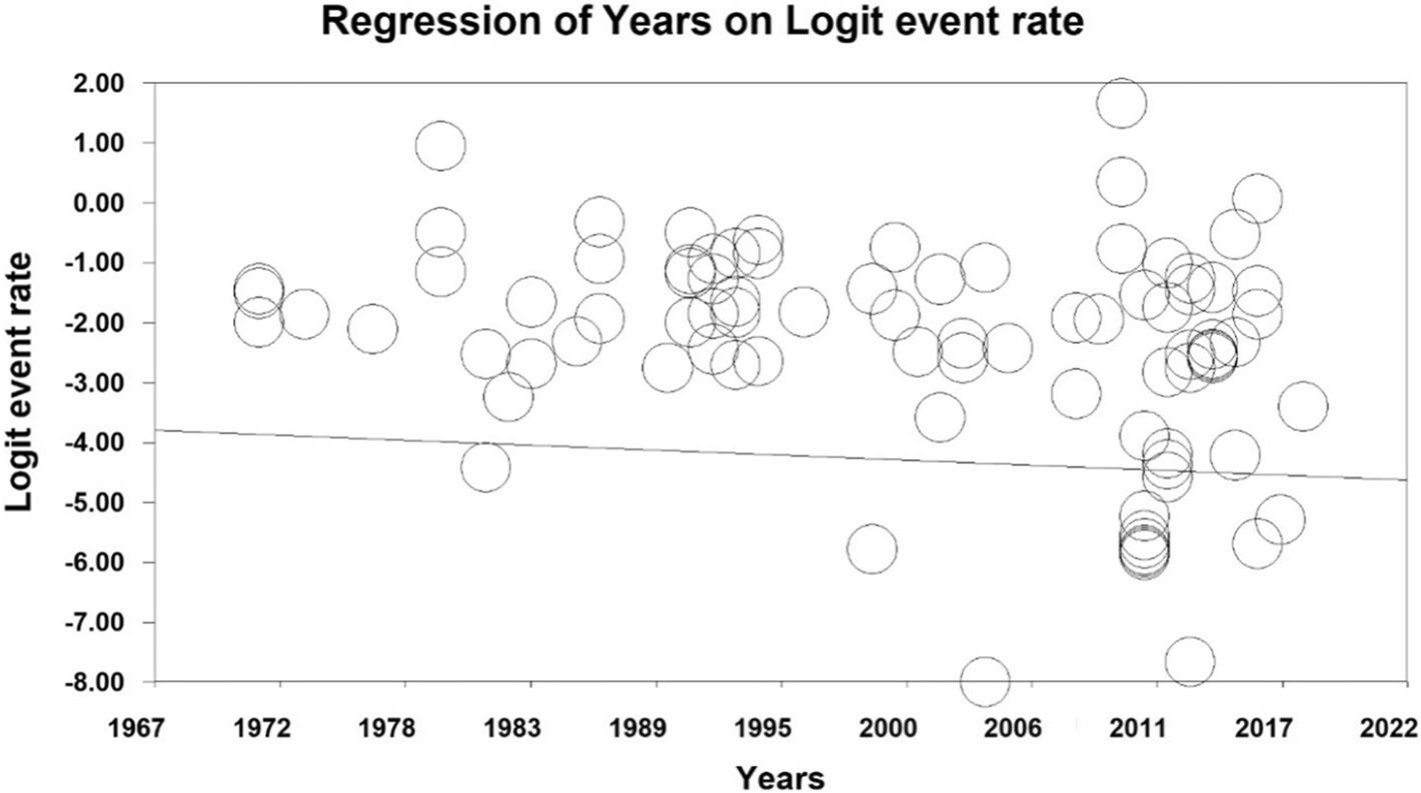
Meta-regression diagram of the prevalence of Dupuytren disease in the world by year of study

In order to investigate the effects of potential factors affecting the heterogeneity of the prevalence of the Dupuytren disease, meta-regression was used for the two factors of sample size and year of study (Figs. 4 and 5). According to Fig. 4, by increasing of sample size, the prevalence of the Dupuytren in the world decreases, which has a statistically significant difference (*P* < 0.05). Moreover, as reported in Fig. 5, with the increase of the year of the study, the prevalence of the Dupuytren in the world decreases; this difference is also statistically significant (*P* < 0.05).

### Subgroup analysis

Table 2 reports the variations of the prevalence of the Dupuytren disease in different continents. The highest ratio is 17.2% which belongs to Africa (95% CI 13–22.3%). Table 3 reports the prevalence of the Dupuytren disease based on the underlying diseases. These variations are reported in the general population; diabetic population; patients with diabetic type I, diabetic type II and rheumatoid arthritis; and alcoholic patients. The highest rate is among patients diagnosed with type 1 diabetes with 34.1% (95% CI 25–44.6%).

**Table 2:** The prevalence of the Dupuytren in the population of different continents

Continents	Number of articles	Sample size	***I***^**2**^	Begg and Mazumdar test	Prevalence % (95 % CI)
Asia	10	25269	99.3	1.000	15.3 (95 % CI 7.5–28.5)
Europe	56	2176967	99.8	0.012	10.3 (95 % CI 6.4–16.2)
Africa	2	1111	42	-	17.2 (95 % CI 13–22.3)
America	17	4425159	99.8	0.773	2.3 (95 % CI 1.4–3.8)

**Table 3:** Prevalence of Dupuytren disease in the population by underlying diseases

Continents	Number of articles	Sample size	***I***^**2**^	Begg and Mazumdar test	Prevalence % (95 % CI)
Diabetic population	15	10161	97.9	0.843	18.1 (95 % CI 11.8–26.7)
General population	32	5329922	99.9	0.011	6.4 (95 % CI 3.8–10.5)
Diabetic type I	2	88	0	-	34.1 (95 % CI 25–44.6)
Diabetic type II	2	260	3.3	-	25.1 (95 % CI 20.1–31)
Rheumatoid arthritis	2	447	33.1	-	7.5 (95 % CI 4.6–12)
Alcoholic patient	5	693	81.4	0.220	24.0 (95 % CI 17.2–32.3)

## Discussion

Dupuytren is a hand deformity that usually progresses over several years. This condition affects a layer of tissue under the skin of the palm. Tissue knots form under the skin and eventually create a thick cord that can bend one or more fingers. This results in the patient not being able to completely straighten the affected fingers [20]. Dupuytren can make it difficult to use hands to perform certain tasks. Since the thumb and the forefinger are not usually affected, many people do not have much discomfort or inability to perform motor activities such as writing. Yet, with the progression of the disease, the ability to use the hands becomes limited [37]. The risk factors for Dupuytren include aging (Dupuytren usually occurs after the age of 50), gender, smoking and alcohol consumption, diabetes, family history and geographic location [28]. Due to the significance of this disease and the lack of general statistics on its status worldwide, the aim of this study was to determine the prevalence of the Dupuytren disease in the world through a systematic review and meta-analysis study.

According to the present study, the prevalence of Dupuytren disease in the world is 8.2% (95% CI 5.7–11.7%). The highest prevalence of Dupuytren disease was related to the study of Rajendran et al. [21] with 84% and the lowest prevalence was related to the study of Weinstein et al. [74] with 0.3%. In a meta-analysis conducted by Lanting et al. [75], the prevalence of Dupuytren in the Western countries was reported as 6–6.31% [75]. Also, Carloni et al. [76], using a meta-analysis work, reported this rate as 1–5.9% [76]. Our study findings are almost in line with these research works. However, the cause of the minor differences between the present study and these pieces of research can be justified based on the number of articles reviewed in the present study which is higher (85 articles in the present study vs. 17 articles in the study of Carloni et al. [76] and 23 articles in the study of Lanting et al. [75]). Moreover, the present study examined patients with different races and geographical locations worldwide.

Due to the change in the demographics in different countries around the world, it is essential to carefully examine the prevalence of Dupuytren disease to acknowledge policy-makers. This can lead to raise more awareness to the disease’s processes and consequences. Therefore, according to the subgroups analysis based on different continents, the highest rate of the prevalence of Dupuytren is related to the African continent with 17.2% (95% CI 13–22.3%), and the lowest is related to the American continent with 2.3% (95% CI 1.4–3.8%).

According to the subgroup’s analysis of the underlying diseases, the highest prevalence is primarily in patients with type I diabetes with 34.1%. The second highest rate is among patients with type II diabetes with 25.1%. Diabetes is one of the most common metabolic diseases, in which hyperglycemia causes pathophysiological changes in various organs. Diabetes is a disease with systemic involvement which includes musculoskeletal problems such as Dupuytren. This is more common in diabetic patients compared to the general population [77]. Dupuytren disease is somewhat preventable and treatable, but not completely cured [78]. Therefore, diagnosis, prevention and treatment of this complication are essential. It is recommended that musculoskeletal examination is included as part of periodic care in diabetic patients.

Furthermore, according to the subgroup’s analysis of the underlying diseases, the highest prevalence of the disease, after diabetic patients, is related to alcoholic patients (24%). Various studies reveal that the prevalence of Dupuytren in alcoholics is higher than the general population; however, the cause is still not clear [79, 80]. Alcohol consumption and its misuse as a social harm have complex interconnected economic, social and cultural causes and have serious safety and health consequences for the society. Therefore, it requires a comprehensive and unified plan along with cooperation between various governing departments.

The most comprehensive study in terms of sample size is the research work conducted by Nordenskjöld et al. in Sweden [27]. They reported prevalence of Dupuytren disease as 0.5% which differs from the findings of our work. However, it is consistent with the results of meta-regression analysis, which revealed that by increasing the sample size and study year, the prevalence rate in the world decreases. According to the results of meta-regression, with the increase of the study year, the prevalence of the world decreases. This reduction can be associated with appropriate preventive measures such as controlling diabetes and blood sugar, avoiding smoking and alcohol consumption and treatment of liver disease.

Dupuytren disease has considerable negative consequences for the patients. Hence, it is important to take measures to achieve effective or supportive treatments to reduce the symptoms of the disease. In addition, in recent years, studying musculoskeletal conditions have been considered an important issue in health care. Since these studies can provide useful information for health care providers and enhance health and therapeutic interventions to improve the quality of services. Ultimately these could lead to improving the quality of life of the patients [81–83].

### Limitations

It can be highlighted that some samples were not based on random selection which, to some extent, appeared as a limitation. Also, other limitations can be signified such as variations in presenting the findings of articles, implementation methods and inaccessibility of the full text of the papers which are presented at the conferences.

### Implication

The results of this study reveal that the prevalence of Dupuytren disease is high, particularly in alcoholic patients with diabetes. Therefore, the officials of the World Health Organization (WHO) are required to develop measures for the prevention and treatment of this disease.

## Data Availability

Datasets are available through the corresponding author upon reasonable request.

## Abbreviations

WHO: World Health Organization
MCP: Metocarpophalangeal
PIP: Proximal interphalangeal
DIP: Distal interphalangeal
COPD: Chronic obstructive pulmonary disease
RA: Rheumatoid arthritis
SID: Scientific Information Database
PRISMA: Preferred Reporting Items for Systematic Reviews and Meta-Analysis
STROBE: Strengthening the Reporting of Observational Studies in Epidemiology for cross-sectional Study
